# Evaluating Stress Distribution Pattern in Periodontal Ligament of Maxillary Incisors during Intrusion Assessed by the Finite Element Method

**Published:** 2015-12

**Authors:** Parisa Salehi, Alayar Gerami, Amirhosein Najafi, Sepideh Torkan

**Affiliations:** a Orthodontic Research Center, School of Dentistry, Shiraz University of Medical Sciences, Iran.; b Dept. of Orthodontics and Dental Research Center, School of Dentistry, Tehran University of Medical Science, Iran.; c Orthodontist, Orthodontic Research Center, Shiraz University of Medical Sciences, Shiraz, Iran.

**Keywords:** Finite Element Method, Intrusion, Miniscrew

## Abstract

**Statement of the Problem:**

The use of miniscrews has expedited the true maxillary incisor intrusion and has minimized untoward side effects such as labial tipping.

**Purpose:**

The aim of this study was to assess the stress distribution in the periodontal ligament of maxillary incisors when addressed to different models of intrusion mechanics using miniscrews by employing finite element methods. The degree of relative and absolute intrusion of maxillary incisors in different conditions was also evaluated.

**Materials and Method:**

Finite element model of maxillary central incisor to first premolar was generated by assembling images obtained from a three-dimensional model of maxillary dentition. Four different conditions of intrusion mechanics were simulated with different placement sites of miniscrews as well as different points of force application. In each model, 25-g force was applied to maxillary incisors via miniscrews.

**Results:**

In all four models, increased stress values were identified in the apical region of lateral incisor. Proclination of maxillary incisors was also reported in all the four models. The minimum absolute intrusion was observed when the miniscrew was placed between the lateral incisor and canine and the force was applied at right angles to the archwire, which is very common in clinical practice.

**Conclusion:**

From the results yield by this study, it seems that the apical region of lateral incisor is the most susceptible region to root resorption during anterior intrusion. When the minimum flaring of maxillary incisors is required in clinical situations, it is suggested to place the miniscrew halfway between the roots of lateral incisor and canine with the force applied to the archwire between central and lateral incisor. In order to achieve maximum absolute intrusion, it is advised to place miniscrew between the roots of central and lateral incisors with the force applied at a right angle to the archwire between these two teeth.

## Introduction

Correction of deep overbite can be accomplished through different treatment strategies. Nonsurgical methods include either extrusion of posterior teeth or intrusion of anterior teeth or both. The decision depends on several factors such as incisor display, smile line and vertical dimension of the patient’s face.[1-3] In patients with great interlabial gap and gummy smile, intrusion of maxillary incisor is the treatment of choice.[4]

Conventional methods for incisor intrusion include 2×4 appliances such as utility arch, Burstone intrusion arch or reverse curved arches.[5-11] Some adverse side effects such as labial tipping of upper anterior teeth might result. Therefore, anterior teeth protrusion would be the actual result of the intrusion arch mechanics in lieu of true incisor intrusion.[3, 11]

The use of temporary anchorage devices (TADs) has facilitated different types of tooth movements.[12-16]Miniscrews offer several advantages such as immediate loading, easy insertion and removal process, and several placement sites along with minimal expenses for the patient.[17] Previous investigations have reported successful intrusion of maxillary anterior teeth using miniscrews when relatively light forces were used.[17-19]It has been shown that true maxillary incisor intrusion would be possible with the use of miniscrews[18-19] and untoward side effects such as labial tipping has been reduced to a minimum, unlike the situations that conventional intrusion mechanics are employed.[20] Nevertheless, the placement site of the miniscrew as well as the point of force application play a very important role in the displacement of the incisor segment and therefore must be selected carefully.[21]

It has been well documented that the principal factor in the initiation of orthodontic tooth movement is the biologic changes that take place in the PDL. However, due to the complex structure of the periodontal tissue, proper assessment of stress profiles and the resultant changes in it after application of orthodontic forces is difficult.[22]

In order to assess the biomechanical factors such as stress, strain and displacement in the teeth as well as the surrounding structures genuinely, finite element method (FEM) was introduced to the field of biomechanical studies.[23] This method is used to evaluate complex structures biomechanically by dividing them into smaller pieces namely element and then carefully analyzing the assembled elements to form a mathematical model of a certain structure.[24] The results of FEM analysis not only depends on the force magnitude, but also the deformation of the structures as well as their geometries.[25] Since this method has the ability of assessing different shapes with different properties, it seems to be a viable method in analyzing the changes in the periodontal ligament and the surrounding structures following tooth movement.[26]

The distribution of stress on the central incisor root with different morphologies under different types of orthodontic tooth movements has been evaluated.[27] The aim of this study was to use FEM to assess the stress profiles in the periodontal ligament of maxillary incisors during orthodontic intrusion via miniscrews. This can lead to a better understanding of the best site of placement of the miniscrews and the point of force application for the desired type of tooth movement.

## Materials and Method

**Figure 1:**
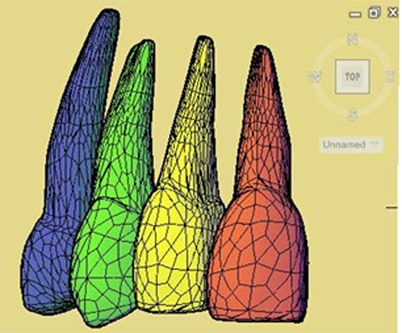
The three-dimensional model of the maxillary central to first premolar

The central incisor, lateral incisor, canine and first premolar were extracted from a 3D computer model of the maxillary teeth on an ovoid arch (3-D studios.com™). Since the model was in the format of DXF, the teeth were defined as surfaces and thus needed to be turned into solid bodies. This process was carried out via the Auto Cad software (version 2011), and the format was changed into SAT which made it possible to work within the ANSYS environment, version 12 (Canonsburg; Pa) (Figure 1).

The tip and torque degree of each tooth is outlined in Table 1.

**Table 1:** The prescribed tip and torque for maxillary teeth in the 3-D model (in degrees)

**Tooth**	**Tip (degrees)**	**Torque (degrees)**
Central	5	14
Lateral	8	7
Canine	10	-3
First premolar	0	-7

The periodontal membrane was supposed to have a uniform thickness (0.25 mm) with a linear behavior,[24] even though this did not truly reflect the complex behavior of PDL, but this method has been shown to be valid for orthodontic loading in FEM studies.[28-29] During the next step, cortical bone was added to the 3D model. The thickness of the cortical bone started from 1 mm at the crest of alveolar bone and increased to 2 mm toward the apical area of the roots of the teeth.[30]

The three-dimensional models of 0.018"×0.025" standard Edgewise brackets (3M Unitek; Monrovia, CA) were fabricated on the labial surfaces of the central and lateral incisors with a 4-mm distance from the incisal edge of the central incisor and 3.5 mm form the lateral incisor. The brackets were attached to the teeth so that the midpoint of the bracket would coincide with the midpoint of the labial surfaces of the incisors. The inter-bracket distance was 7 mm.[31] Afterwards, the 3D model of the arch wire (0.016×0.022) was also designed and placed inside the bracket slots.

**Figure 2:**
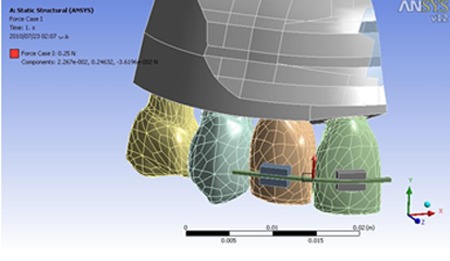
The finite element model prior to loading

The entire assembly of the 3D model was imported into the ANSYS workbench software (version 12.0.1; Canonsburg, Pa) (Figure 2).

All the materials in the finite element analysis were assumed to be homogenous, isotropic, and linearly elastic. The interfaces of the bracket-tooth, bracket-archwire, and bone-miniscrew were defined as fully bonded surfaces.[24] All the mechanical properties of the components of the model are represented in Table 2.

**Table 2:** Mechanical properties of different materials in the finite element model

**Material**	**Young’s Modulus**	**Poisson’s Ratio**
Tooth	2×10^4^	0.3
PDL	0.68	0.49
Alveolar Bone (Mean)	2×10^3^	0.3
Stainless Steel	2.1×10^5^	0.3

In order to evaluate the stress profiles in the periodontal ligament as well as the rate of vertical displacement of the incisal edges of the incisors, miniscrews were reconstructed in the finite element model in four different conditions. In all four different conditions, miniscrews were placed at a 4-mm distance from the crest of the alveolar bone. This distance was selected based on the fact that the mucogingival junction of the upper anterior teeth is located 4-5 mm from the gingival margin.[32] Lim* et al.* also suggested the miniscrews to be placed 6 mm apical to the crest of the alveolar bone.[33] In the sagittal plane, the miniscrews were fabricated 1 mm external to the bone to represent the thickness of the gingiva.[32]

In the first model, the miniscrew was placed halfway between the roots of central and lateral incisors and the force was applied at a right angle to the archwire between these two teeth.

In the second model, the miniscrew was placed halfway between the roots of lateral incisor and canine and the force was applied to a point on the archwire between the central and lateral incisors.

In the third model, the miniscrew was placed halfway between the roots of lateral incisor and canine and the force was applied at a right angle to the archwire between the lateral incisor and canine.

In the fourth model, the miniscrew was placed halfway between the roots of central and lateral incisors and the force was applied to a point on the archwire between lateral incisors and canine. 

In each model, the finite element analysis was realized by applying a total of 25 g force from the miniscrew to the arch wire.[1]

The total number of elements used in this finite element model was 67780 elements and 119583 nodes. Boundary conditions were assigned to the nodes on the cortical bone above the apical area of the teeth as zero displacement in all directions. All the other nodes had 3 translational degree of freedom (X: mesiodistal; Y: vertical; Z: vestibulopalatinal).

**Statistical analysis**

Descriptive analysis was used to analyze the degree of displacement in the Y plane (vertical) as well as Z plane (vestibulopalatinal). Using the finite element analysis, the stress profiles in the periodontium of the central and lateral incisors were also defined. 

## Results

**Figure 3:**
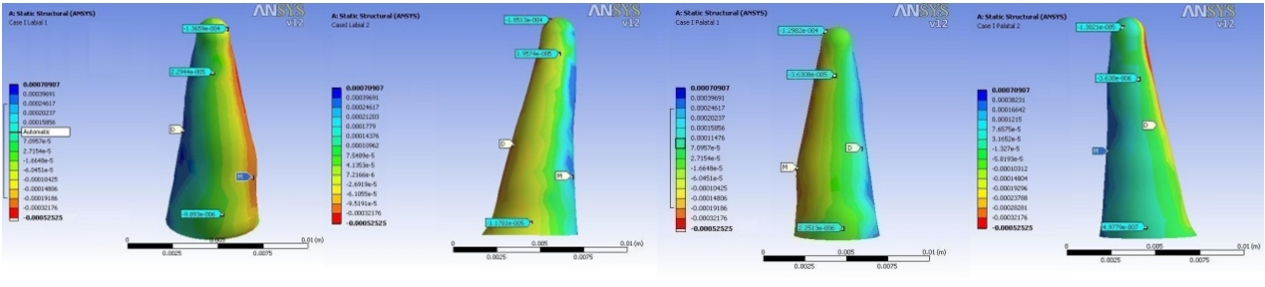
Von Mises stress distribution along the root surfaces of the central and lateral incisors in the first model (from left to right: central: buccal, lateral: buccal, central: palatal, lateral: palatal; M: mesial, D: distal)

**Figure 4:**
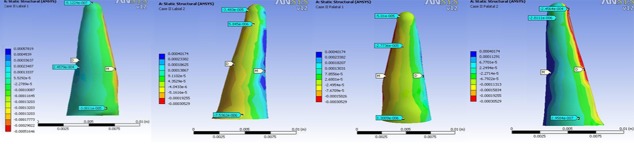
Von Mises stress distribution along the root surfaces of the central and lateral incisors in the second model (from left to right: central: buccal, lateral: buccal, central: palatal, lateral: palatal; M: mesial, D: distal)

**Figure 5:**
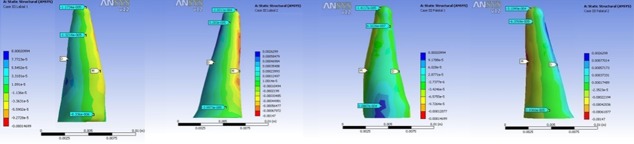
Von Mises stress distribution along the root surfaces of the central and lateral incisors in the third model (from left to right: central: buccal, lateral: buccal, central: palatal, lateral: palatal; M: mesial, D: distal)

**Figure 6:**
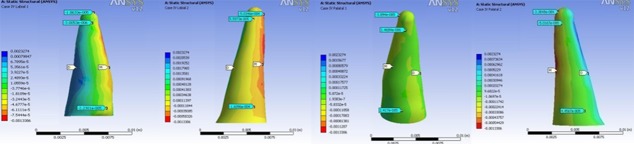
Von Mises stress distribution along the root surfaces of the central and lateral incisors in the fourth model (from left to right: central: buccal, lateral: buccal, central: palatal, lateral: palatal; M: mesial, D: distal)

In all models, the highest stress magnitude was produced near the apex of the lateral incisor on the palatal surface. Figures 3 to 6 demonstrate the Von Mises stress distribution along the root surfaces of the central and lateral incisors. Tables 3 to 10 outline the stress distribution pattern of the central and lateral teeth based on their respective models.

**Table 3:** Maximum stress distribution in PDL of the central teeth in the first model

**PDL of the Central Teeth (First Model)**			
Mesial Surface	Labial Surface	Distal Surface	Palatal Surface
1/3 Apical, Apex	1/3 Marginal	1/3 Marginal	1/3 Apical, Apex

**Table 4:** Maximum stress distribution in PDL of the lateral teeth in the first model

**PDL of the Lateral Teeth (First Model)**			
Mesial Surface	Labial Surface	Distal Surface	Palatal Surface
1/3 Marginal	1/3 Marginal	1/3 Apical, Apex	1/3 Apical, Apex

**Table 5:** Maximum stress distribution in PDL of the central teeth in the second model

**PDL of the Central Teeth (Second Model)**			
Mesial Surface	Labial Surface	Distal Surface	Palatal Surface
1/3 Apical, 1/3 Middle	1/3 Marginal	1/3 Marginal	1/3 Apical, Apex

**Table 6:** Maximum stress distribution in PDL of the lateral teeth in the second model

**PDL of the Lateral Teeth(Second Model)**			
Mesial Surface	Labial Surface	Distal Surface	Palatal Surface
1/3 Marginal	1/3 Marginal	1/3 Apical, 1/3 Middle, Apex	1/3 Apical, Apex

**Table 7:** Maximum stress distribution in PDL of the central teeth in the third model

**PDL of the Central Teeth (Third Model)**			
Mesial Surface	Labial Surface	Distal Surface	Palatal Surface
1/3 Apical, 1/3 Middle, Apex	1/3 Marginal, 1/3 Middle	1/3 Marginal	1/3 Apical , Apex

**Table 8:** Maximum stress distribution in PDL of the lateral teeth in the third model

**PDL of the Lateral Teeth(Third Model)**			
Mesial Surface	Labial Surface	Distal Surface	Palatal Surface
1/3 Apical, 1/3 Middle, Apex	1/3 Marginal	1/3 Marginal	1/3 Apical , Apex

**Table 9:** Maximum stress distribution in PDL of the central teeth in the fourth model

**PDL of the Central Teeth (Fourth Model)**			
Mesial Surface	Labial Surface	Distal Surface	Palatal Surface
1/3 Apical, Apex	1/3 Marginal, 1/3 Middle	1/3 Marginal, 1/3 Middle	1/3 Apical , Apex

**Table 10:** Maximum stress distribution in PDL of the lateral teeth in the fourth model

**PDL of the Lateral Teeth (Fourth Model)**			
Mesial Surface	Labial Surface	Distal Surface	Palatal Surface
1/3 Apical, Apex	1/3 Marginal	1/3 Marginal	1/3 Apical, Apex

Vertical displacement of the nodes at the incisal edge of the incisors, relative intrusion and absolute intrusion of the incisors in the four models are outlined in Table 11. In order to compare the values for absolute and relative intrusion in the four models in Table 11 more readily, the smallest value was considered as the base (100) and the rest of the values were ranked accordingly. The results are presented in Table 12.

**Table 11:** Vertical displacement of the nodes at the incisal edge of maxillary incisors, the amount of relative and absolute intrusion in four models of loading (the values are presented in micrometer)

		**First model**	**Second model**	**Third model**	**Fourth model**
Central incisor	Relative intrusion	3.45×10 ^-1^	1.88×10^-1^	1.140×10^-1^	2.050 × 10 ^-1^
	Labial displacement of the incisal edge	2.919×10^-1^	9.184×10^-2^	1.610×10^-1^	3.260 × 10 ^-1^
	Absolute intrusion	1.75×10^-1^	1.032×10^-1^	2.994×10^-2^	7.566 × 10 ^-2^
Lateral incisor	Relative intrusion	3.340×10^-1^	2.630×10^-1^	1.700×10^-1^	3.11 × 10 ^-1^
	Labial displacement of the incisal edge	2.573×10^-1^	9.376×10^-2^	2.006×10^-1^	3.628 × 10 ^-1^
	Absolute intrusion	1.167×10^-1^	5.985×10^-2^	8.27×10^-3^	4.41 × 10 ^-2^

**Table 12:** Comparison of labial displacement, relative and absolute intrusion of the maxillary incisors in the four models. 100=8.27×10-3

		**First model**	**Second model**	**Third model**	**Fourth model**
Central incisor	Relative intrusion	4171	2273	1378	2478
	Labial displacement of the incisal edge	3529	1110	1947	3942
	Absolute intrusion	2116	1245	356	914
Lateral incisor	Relative intrusion	4038	3180	2055	3760
	Labial displacement of the incisal edge	3107	1133	2426	4388
	Absolute intrusion	1410	722	100	533

In all four models of loading except for the first model, the labial displacement of the lateral incisor (Z plane) was more than the central incisor. The maximum labial displacement was observed in the fourth model followed by the first, third, and second models, respectively (4388 in the fourth model vs. 1133 in the second model). 

In all four models except for the first model, the degree of relative intrusion (Y plane, apical displacement of the incisal edge) observed in the lateral incisor was higher than the central incisor. The maximum relative intrusion values were reported in the first model, followed by the fourth, second and third models, respectively (the maximum relative intrusion of the lateral incisor were 4038 vs. 2055 for the first and third models, respectively).

In all four models, the amount of absolute intrusion (Y plane, apical displacement of the apex) of the central incisor was more than the lateral incisor. The maximum absolute intrusion values were identified in the first model followed by the second, fourth and third models respectively (2116 in the first model compared with 356 in the third model).

As it can be deduced from Table 11, in all four models except for the second one in the central incisor case, the values recorded for the labial displacement of each tooth was higher than the values recorded for the absolute intrusion of the same tooth (the values for the labial displacement and absolute intrusion of the central incisors were respectively 3529 and 2116 in the first model, 1947 and 356 in the third model and 3942 and 914 in the fourth model).

## Discussion

Successful intrusion of the anterior teeth without placing adverse side effects on the posterior teeth has been an issue of interest in orthodontic camouflage of gummy smile, vertical maxillary excess patients. Miniscrews have provided the clinicians with an opportunity to obtain the desired intrusion with minimal side effects. However, there are still some obscure data regarding the optimal placement site and the point of force application for each type of tooth movement.

This finite element study was conducted to analyze different patterns of stress distribution in the periodontal ligament of maxillary incisors upon placing intrusion force on them. The degree of displacement in different planes of space was also studied. In order to maximize the accuracy of the results of the study, the cortical bone was added manually to the model with different thicknesses at different levels to better represent the maxillae.[24]

When four different models of the study were compared, it was observed that in the first and second models, root divergence was reported between the central and lateral incisors; as the central incisor experienced an uncontrolled tipping movement of the crown to distal and labial along with intrusion; while, in the lateral incisor, an uncontrolled tipping of the crown was recorded to mesial and labial. On the other hand, in the third and fourth models, a convergence was experienced between the root of the incisors, as both the central and lateral incisor crowns were tipped distally and labially. In order to decrease the undesired mesiodistal crown movement, it can be advocated that the largest possible archwire be fit into the bracket slot (0.018×0.025-in) to minimize the resultant deflection in the archwire. The present study, demonstrated that lateral and central incisors move independently and therefore do not act as a single unit. Reimann *et al.* also disproved the theory of a single center of resistance for the entire anterior segment.[34] Using a larger diameter of the main archwire might decrease the play between the wire and bracket, leading to a more similar center of resistance for both incisors and more similar type of movement in both teeth.[34]

In comparison between the four models regarding the actual value of labial displacement, the minimal labial displacement of the incisal edges of the incisors occurred in the second model, followed by the third, first and fourth models, respectively. What first and fourth models had in common was the miniscrew placement site. Thus, it can be concluded that the closer the miniscrew is placed to the midline, the more labial tipping of the incisors can be expected. In addition, it can be speculated that the placement site of the miniscrew plays a more important role than the point of force application in the resultant labial tipping. Based on the study conducted by Burstone, the intrusive force must be applied to a point between the lateral incisor and canine to decrease the untoward labial tipping of the entire segment.[2, 35-36] Other studies suggested the center of resistance of the anterior segment to be 8-10 mm apical and 5-7 mm distal to the lateral incisor.[2, 35, 37]

In all four models, however, a degree of labial tipping was observed. Therefore, the center of resistance was probably located more distally based on the study of Vanden Bulcke *et al.* who advocated the force to be applied between the canine and first premolar.[38] Polat-Ozsoy *et al.* placed the miniscrew between the lateral incisor and canine; the third model of the present study resembles their model. They reported a minimal increase in the proclination of the upper incisors which was not statistically significant.[19]

It should be considered that the center of resistance depends on several factors such as the surrounding bone, morphology and the length of the roots, and the inclination of the upper teeth. Therefore, the optimal point for the applied force depends on the individual variations.[37]

In all four different models, Von Mises stress distribution was recorded to be the highest in the apex of the lateral incisor. This can be attributed to the smaller surface area and geometry of the apex of the lateral incisor.[39] This area should be considered as a point high ly prone to resorption in intrusive mechanics. This result was in consistence with previous histological findings that reported the concentration of the cell-free area and hyalinization zones at the apex which corresponded to higher stress in that area.[40] It was also reported that a substantial amount of stress was created in the cementum and PDL as a whole.[41]

Since the stress is the principal cause of the biomechanical response, it is expected the maximum absolute intrusion be recorded in the lateral incisor as well. However, the maximum absolute intrusion was observed in the central incisor in all four models. This can be explained by the fact the mean movement of the apex of lateral incisor in all three planes of space was greater than the central incisor. Absolute intrusion, on the other hand, is merely a movement in vertical direction. 

The maximum absolute intrusion of the incisors was defined in the first model, followed by the second, fourth, and third models, respectively. Since the point of force application was the same in the first and second models, it can be speculated that the point of force application was a more important determinant in the degree of absolute intrusion than the placement site of the miniscrew. The higher absolute intrusion of the first model compared with the second one can be attributed to the bigger vertical vector in the first model. An interesting result was that the minimum absolute intrusion observed in the third model which is the most common method of force application for intrusion mechanics in clinical practice.

The absolute intrusion of the central incisor in all four models was more than the lateral incisor, while the opposite was true regarding the relative intrusion in all four models except for the first model. Therefore, it can be concluded that the labial tipping of the lateral incisor was greater than the central incisor in the three aforementioned models.

Since the present study did not consider the individual variations, it should be kept in mind that these variations require an individualized mechanics of force system for each person. As stated by Kamble *et al.*, central incisors with deviated root morphologies are more prone to root resorption following different types of orthodontic tooth movement; thus, highlighting the effect of individual variation.[27] The finite element analysis only considers the initial tooth movements and stress distributions in the periodontal ligament; as the process of tooth movement progresses, the force system, stresses and biologic responses of each individual might change. There were other limitations in the methodology of this study. The values used for the mechanical properties of different tissues were constant in this study, but this is not the case in clinical practice. The periodontal ligament was also considered to have a uniform and homogenous thickness which does not reflect true clinical situations. Therefore, the results might not be applicable to each individual.[24]

## Conclusion

The apical region of lateral incisor experienced the highest stress levels. This site should be considered as the most susceptible site to resorption. The maximum absolute and relative intrusion was obtained in the first model. Therefore, in clinical situation in which intrusion along with some degree of labial tipping is acceptable, the first model of force application can be suggested. The second model represented the best combination of intrusion with a minimal labial tipping of the crown of the incisors. Hence, in clinical situations in which the minimal labiolingual displacement is desirable, this model can be suggested. Midline deviation to the screw side was not mentioned. The fourth model is the least acceptable model of force application since it provides the maximum proclination of the incisors and some degree of absolute intrusion. The third model which is a common method of force application in clinical practice, produced the minimum absolute intrusion, but was acceptable regarding the degree of labial tipping of the incisors. 
